# Remotely sensed estimation of total iron content in soil with harmonic analysis and BP neural network

**DOI:** 10.1186/s13007-021-00812-8

**Published:** 2021-11-12

**Authors:** Xueqin Jiang, Shanjun Luo, Shenghui Fang, Bowen Cai, Qiang Xiong, Yanyan Wang, Xia Huang, Xiaojuan Liu

**Affiliations:** 1 School of Remote Sensing and Information Engineering, Wuhan University, Wuhan, 430079 China; 2 Lab of Remote Sensing for Precision Phenomics of Hybrid Rice, Wuhan University, Wuhan, 430079 China

**Keywords:** Total iron content, Harmonic analysis, Wavelet packet transform, Principal component analysis, BP neural network

## Abstract

**Background:**

The estimation of total iron content at the regional scale is of much significance as iron deficiency has become a routine problem for many crops.

**Methods:**

In this study, a novel method for estimating total iron content in soil (TICS) was proposed using harmonic analysis (HA) and back propagation (BP) neural network model. Several data preprocessing methods of first derivative (FD), wavelet packet transform (WPT), and HA were conducted to improve the correlation between the soil spectra and TICS. The principal component analysis (PCA) was exploited to obtained three kinds of characteristic variables (FD, WPT-FD, and WPT-FD-HA) for TICS estimation. Furthermore, the estimated accuracy of three BP models based on these variables was compared.

**Results:**

The results showed that the BP models of different soil types based on WPT-FD-HA had better estimation accuracy, with the highest R^2^ value of 0.95, and the RMSE of 0.68 for the loessial soil. It was proved that the characteristic variable obtained by harmonic decomposition improved the validity of the input variables and the estimation accuracy of the TICS models. Meanwhile, it was identified that the WPT-FD-HA-BP model can not only estimate the total iron content of a single soil type with high accuracy but also demonstrate a good effect on the estimation of TICS of mixed soil.

**Conclusion:**

The HA method and BP neural network combined with WPT and FD have great potential in TICS estimation under the conditions of single soil and mixed soil. This method can be expected to be applied to the prediction of crop biochemical parameters.

## Background

Iron is an indispensable trace element for plants, whose content in soils largely relies on the pH value and the water content, and is influenced by root respiration, soil microbial activity, leaching, and erosion [1]. Accurate estimation of the total iron content in soil (TICS) is helpful for agronomists to assess soil conditions, which is also the key to ensure the healthy growth of crops. Therefore, rapid and precise prediction of TICS has an important practical value for precision agriculture [2, 3].

The traditional determination methods of TICS include the combination of field sampling and laboratory measurement, assisted by atomic absorption spectrometry [4] and o-phenanthroline colorimetry [5]. Although a high accuracy was obtained, these methods were time-consuming and costly on a large-area application. In recent years, hyperspectral remote sensing (HRS) technology has the advantages of high resolution, fast speed, and high accuracy, which makes it possible to estimate the TICS in a large area quickly and efficiently [6]. HRS is one of the frontier remote sensing technologies since it provides continuous spectral information about each feature of the research object [7]. Several scholars have used HRS to predict nitrogen content [8], water content [9], and heavy metal content in soils [10]. They found that spectral preprocessing plays an important role in quantitative inversion and estimation.

At present, the TICS retrieving accuracy of HRS is limited by using traditional preprocessing methods due to the low iron contents and various existing forms in soils. For example, Bendor and Banin established a multiple linear regression model to predict the TICS by using 1075, 1025, and 425 nm spectral bands, with the highest R^2^ of 0.76 [11]. Subsequently, a large number of researchers applied first and second derivative [12], reciprocal and logarithm [13], continuum removal methods [14] to expand spectral differences and reduce noise interference, thereby increasing the correlation between spectral data and TICS. This can be seen in the case that Guo et al. analyzed the correlation between different spectral forms and the content of iron oxide in soil by different spectral transformations, and the highest inversion accuracy was 0.93 [15].

The characteristic bands were used to serve as the characteristic variables in many studies. However, the soil spectral curve is a comprehensive manifestation of the interaction and superposition of various substances, thus the determination of the characteristic bands is not only difficult but also highly uncertain. Due to the complexity of soil compositions, the interference of other components will lead to the signal-to-noise ratio reduced under the conditions of low TICS in soils. Therefore, the selection of appropriate spectral denoising methods [such as first derivative (FD), wavelet transform, wavelet packet transform (WPT), filtering, and average weighting] is of great importance. The wavelet transform has a strong ability to remove noise [16]. When it was applied to soil spectral data analysis, the spectral signal can be decomposed into sub-signals with different frequencies. It can effectively use the overall structural characteristics of spectral information, extract the weak information hidden in the spectral signal, and search for the best combination of sub-signal components to estimate the TICS. Gu et al. found that soil organic matter content can be retrieved using the high-frequency coefficients created with the wavelet transform and random forest algorithm [17].

Given the above descriptions, new methods for estimating TICS still need to be explored. Harmonic analysis (HA) was proposed to transform the time domain of the preprocessed spectral data into the frequency domain [18]. Harmonic decomposition can suppress or eliminate the background noise of ground objects and achieve the effect of data compression. The best harmonic component obtained by harmonic decomposition can be used as the characteristic variable to construct the inversion model of TICS. The inversion accuracy of TICS depends on the selection of the inversion model and characteristic variables. Quantities of studies have shown that the statistical analysis methods can be applied to the hyperspectral inversion of TICS, and the back propagation (BP) neural network can deal with the nonlinear situation well in the estimation of TICS as a result of its strong self-learning ability [19]. With the denoising ability of FD and WPT, and the frequency domain information provided by HA, the variables estimated TICS with high precision can be provided. Meanwhile, the accuracy of TICS prediction can be expected to be improved by using the dimensionality reduction of principal component analysis (PCA) and the ability of BP nonlinear learning. As the significant differences in physical and chemical properties of different types of soils, the study of soil spectral characteristics is relatively complex. Therefore, it is meaningful to carry out the estimation of total iron content in different types of soils.

In this paper, the hyperspectra of different types of soils were remotely measured from ground platforms. Meanwhile, various preprocessing technologies (mainly include WPT, FD, and HA) and PCA were employed for dimensionality reduction and feature variable extraction. Then the BP neural network models for TICS estimation of single soil and mixed soils were constructed. Our objectives are (1) to compare and evaluate several widely used characteristic variables for TICS estimation; (2) to analyze the improvement of the retrieving accuracy of TICS by the characteristic variables derived from HA, and (3) to apply the characteristic variables obtained by HA to three different types of soil to explore the optimal characteristic variables for TICS estimation of different soil types.

## Materials and methods

### Study area

**Fig. 1:**
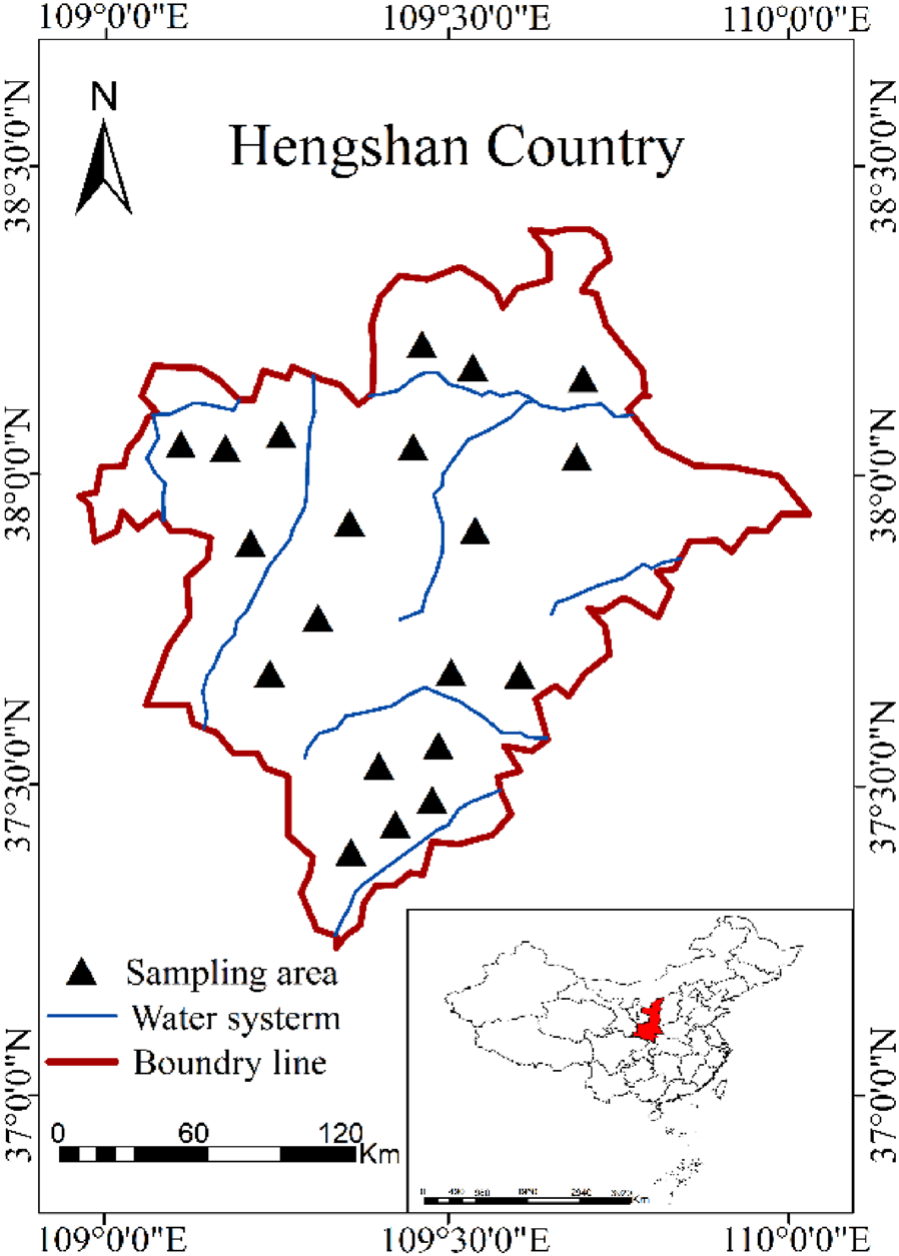
Study area location and sampling points distribution

The field sampling experiments were conducted in Hengshan County, Shaanxi Province, China (37°22′N–38°14′N, 108°65′E–110°02′E), which belongs to the hilly and gully region of the Loess Plateau in the upper and middle reaches of the Yellow River with a total area of 4333 km^2^. Hengshan County was seriously affected by desertification due to the proximity to the Mu Us Desert. It belongs to the semi-dry continental monsoon climate, with an annual average temperature of 8.6 ℃ and average annual precipitation of 399 mm. The soil of this area is mainly composed of loessial soil and sandy soil. The difference in TICS was caused by the above comprehensive factors. The study area (Fig. 1) is conducive to estimating TICS in the districts with rich soil types. Taking Hengshan County as the study area, the occasionality and limitation caught by a single soil type can be avoided, which makes the research results more popularized and adaptable.

### Measurements of total iron content in soil

The soil sampling of TICS was performed in the flat terrain and bare soil areas. Two types of soil (loessial soil and sandy soil) were collected and four or five representative survey points in each sampling area were selected. At each sampling point, a sample of surface soil (about 5 cm) was dug up and put into a plastic bag. A total of 84 soil surface samples were collected, including 51 loessial soil samples and 33 sandy soil samples (Fig. 1; Table 1). All the collected samples were encapsulated and sent to be tested. The atomic absorption method was adopted to measure the TICS.

**Table 1:** Descriptive statistics of total iron content in soil

Soil types	Samples	TICS (g/kg)				
		Min.	Max.	Mean	SD	CV (%)
Loessial soil	51	15.42	27.51	23.26	2.28	9.82
Sandy soil	33	7.20	27.18	19.20	4.60	23.98
Mixed soil	84	7.20	27.51	21.66	3.93	18.13

### Measurements of soil spectral reflectance

The soils were ground and then screened by a 2 mm diameter sieve to obtain the samples for measuring spectral reflectance. The spectral reflectance measurements of the preprocessed soil samples were conducted on the laboratory conditions in a dark room using an ASD Field Spec FR spectrometer (Analytical Spectral Devices, Inc., USA). The spectrometer collected the hyperspectral reflectance from 350 to 2500 nm with the spectral resolution of 3 nm during the range of 350–1000 nm and 10 nm during the range of 1000–2500 nm. A 50 W halogen lamp (0.5 m from the soil samples) was used as the only light source, and the vertical distance from the soil sample to the probe (field of view: 3°) was approximately 0.2 m. A whiteboard (chemical composition: BaSO_4_) was used for relative radiometric correction. Four spectra were averaged into a single one for each soil sample to decrease the instability of the measurements.

### Data processing and analysis

#### Five-point weighted average

The five-point weighted average method was applied to eliminate random errors. The window with five spectral data points was moved in the spectral data of each sample, and the values of the middle data points in the window were smoothed by the two adjacent data points. The weight of data points decreased gradually with the increase of the distance from the middle point, and the value of the middle point of the window was the weighted average of these five points. The calculation formula is as follows:

1 $$S = (S_{n - 2} /4 + S_{n - 1} /2 + S_{n} + S_{n + 1} /2 + S_{n + 2} /4)/2.5$$

where the S is the five-point weighted average value of the spectral data. Among the five spectral data, S_n−2_, S_n−1_, S_n+1_, S_n+2_ are the first, second, fourth, and fifth spectral data, respectively.

#### First derivative of spectra

The first derivative (FD) was performed after a five-point weighted average operation to eliminate some linear background and noise of original spectral data [20]. The formula is shown below:

2 $$R^{\prime}(\lambda_{i} ) = {{\left[ {R(\lambda_{i + 1} ) - R(\lambda_{i - 1} )} \right]} \mathord{\left/ {\vphantom {{\left[ {R(\lambda_{i + 1} ) - R(\lambda_{i - 1} )} \right]} {(\lambda_{i + 1} - \lambda_{i - 1} )}}} \right. \kern-\nulldelimiterspace} {(\lambda_{i + 1} - \lambda_{i - 1} )}}$$

where R′(λ_i_) represents the FD spectral reflectance of wavelength λ_i_. R is the original spectral reflectance.

#### Wavelet packet transform

Wavelet packet transform (WPT) was utilized to precisely decompose and reconstruct high-frequency information (HFI), and to remove the noise of HFI as well. WPT based on the wavelet transform is superior to the wavelet transform in decomposing and reconstructing HFI [21, 22]. The results of information processing do not exit redundancy and omission, so it is more conducive to spectral denoising and original information preservation. In this study, we decomposed and reconstructed the spectral data according to the four steps: (1) wavelet packet analysis of spectra using Db10 as wavelet generating function to decomposed the multi-layer WPT of spectra with noise [23]. (2) calculating the optimal wavelet packet basis of WPT decomposition according to the principle of minimum cost. (3) quantifying the wavelet packet coefficients by selecting the soft threshold with good continuity. (4) reconstruction of WPT spectral information based on the optimal wavelet packet basis and the quantitative optimal wavelet packet decomposition coefficients to obtain the spectra of WPT noise reduction. Finally, WPT spectra were processed by FD to obtain the WPT-FD spectral data to compare with the FD data.

#### Harmonic analysis

Abundant issues of noise and redundancy still existed after the above operations. Harmonic analysis (HA) was proposed to transform the time domain into the frequency domain by taking the processed spectral data (WPT-FD spectra) as a sequence signal. HA, firstly proposed by Jakubauskas et al. was mainly employed in power system harmonic monitoring [24]. In this study, spectral data can be decomposed into a series of harmonic energy characteristic parameters by harmonic decomposition. The harmonic theory was used to express a time-series function f(t) in the form of sine or cosine wave (harmonic) superposition. Namely, any time-series f(t) about time t can be expressed by several sine or cosine wave superpositions. The hyperspectral reflectance of each soil sample can be served as a continuous function (during the range of wavelength). When using HA to process spectral data, the spectral curve composed of N bands can be regarded as a function with its cycle of N. Spectral HA decomposition is to express the spectral curve of each soil sample as the sum of a series of superimposed sine and cosine waves composed of some energy characteristic components such as harmonic remainder (A_0_/2), amplitude (A_h_, B_h_, and C_h_), and phase (φ_h_). A group of spectra consisting of N bands is expressed as V(k)  =  (v_1_, v_2_, …, v_N_), and the spectral reflectance of each band is marked as v_k_ (k  =  1, 2, …, N). The harmonic decomposition expansion formula of h-times HA is as follows.

3 $$\begin{gathered} V(k) = \frac{{A_{0} }}{2} + \sum\limits_{h = 1}^{\infty } {[A_{h} \cos (2\pi hk/N) + B_{h} \sin (2\pi hk/N)]} \hfill \\ {\kern 1pt} {\kern 1pt} {\kern 1pt} {\kern 1pt} {\kern 1pt} {\kern 1pt} {\kern 1pt} {\kern 1pt} {\kern 1pt} {\kern 1pt} {\kern 1pt} {\kern 1pt} {\kern 1pt} {\kern 1pt} {\kern 1pt} {\kern 1pt} {\kern 1pt} {\kern 1pt} {\kern 1pt} {\kern 1pt} {\kern 1pt} {\kern 1pt} {\kern 1pt} {\kern 1pt} {\kern 1pt} = \frac{{A_{0} }}{2} + \sum\limits_{h = 1}^{\infty } {C_{h} \sin (2\pi hk/N + \varphi_{h} )} \hfill \\ \end{gathered}$$

After the h-th harmonic decomposition of V(k), the harmonic characteristic components are calculated.

4 $$A_{0} /2 = \frac{1}{N}\sum\limits_{k = 1}^{N} {v_{k} }$$

5 $$A_{h} = \frac{2}{N}\sum\limits_{k = 1}^{N} {v_{k} \cos (2\pi hk/N)}$$

6 $$B_{h} = \frac{2}{N}\sum\limits_{k = 1}^{N} {v_{k} \sin (2\pi hk/N)}$$

7 $$C_{h} = \sqrt {(A_{h}^{2} + B_{h}^{2} )}$$

8 $$\varphi_{h} = \tan^{ - 1} (A_{h} /B_{h} )$$

where h (h  = 1, 2, 3, …) is the number of harmonic decomposition. When h  = 1, it is the component of the fundamental wave. A_0_/2 is the harmonic remainder and C_h_sin(2πhk/N  +  φ_h_) is the harmonic component of h times. A_h_, B_h_, C_h_, and φ_h_ are the cosine amplitude, sine amplitude, harmonic component amplitude, and the phase of harmonic component of h-th harmonic decomposition, respectively. A_0_/2, C_h_, and φ_h_ reflect the mean energy of each band, the energy change of different bands, and the band position where the energy appears amplitude.

The low order harmonics contain the main energy characteristics of the spectra, and the high order harmonics are generally mixed with noise information. The amplitude and phase, carrying objects band energy and radiation peak position information, reflect the local feature information of spectra. Therefore, harmonic decomposition can not only suppress or eliminate background noise but also highlight the spectral characteristics of objects with low-order harmonic components to achieve the effect of data compression. In this study, the total number of bands in harmonic decomposition is 150. The WPT-FD spectra were used to obtain the WPT-FD-HA spectral data by the HA method.

#### Principal component analysis

Principal component analysis (PCA) was widely used in data feature extraction, compression, and dimensionality reduction [25]. The PCA method was used to transform the extracted data (including FD, WPT-FD, and WPT-FD-HA data). On the premise of retaining as much information as possible, the correlation between the data was eliminated and the characteristic variables were obtained. In a principal component inversion, the principal components whose cumulative variance contribution rate more than 90% after PCA were selected as the inversion parameters [26].

### Back propagation model and accuracy evaluation

In spectral analysis, the back propagation (BP) neural network is an important pattern recognition method, which is suitable for solving some complex mapping problems and has a good effect on complex non-linear prediction and inversion. In this study, the BP model was used to estimate the TICS. The BP neural network consists of three layers: input layer, hidden layer, and output layer [27]. When it talks to the BP neural network estimation, the retrieving speed will increase and the amount of calculation will be decreased by reducing the number of characteristic variables. Therefore, the PCA method was used as the input layer of the BP neural network to improve inversion efficiency.

The topological structure of the BP model used in this study was 5—3—1. The number of nodes of the input layer, hidden layer, and output result layer was set as 5, 3, and 1, respectively. The number of network training iterations was 2000, the learning rate was 0.01, the additional momentum factor was 0.9, and the learning error was 0.001. The above operation was realized based on the software of MATLAB 2018a (MathWorks, Inc., Natick, MA, USA).

In the process of BP model establishment, 35 and 20 groups from 51 groups of loessial soil and 33 groups of sandy soil samples were selected as training samples and the remaining 16 and 13 groups of samples as testing samples. Then we randomly selected 45 groups of samples from 84 groups of mixed soils as the training samples and the remaining 39 groups of sample data as testing samples.

Prior to the PCA operation, a preliminary selection of characteristic bands was made. To retain enough useful information and avoid redundancy, 150 bands with a correlation coefficient greater than 0.55 with TICS were selected as characteristic bands in this paper. These bands will then be further screened using PCA.

The prediction accuracy of the models was determined by the parameters of the coefficient of determination (R^2^), the root mean square error (RMSE), and the mean absolute error (MAE). The high R^2^, low RMSE and MAE values indicate good estimation effects.

9 $$RMSE = \sqrt {\sum\limits_{i = 1}^{n} {{{(y_{i} {\kern 1pt} - {\kern 1pt} \hat{y}_{i} )^{2} } \mathord{\left/ {\vphantom {{(y_{i} {\kern 1pt} - {\kern 1pt} \hat{y}_{i} )^{2} } n}} \right. \kern-\nulldelimiterspace} n}} }$$

10 $$MAE{\kern 1pt} {\kern 1pt} {\kern 1pt} { = }{\kern 1pt} {\kern 1pt} {\kern 1pt} \frac{1}{{\text{n}}}\sum\limits_{i = 1}^{n} {\left| {y_{i} {\kern 1pt} - {\kern 1pt} \hat{y}_{i} } \right|}$$

where y_i_ is the measured value, $\hat{y}_{i}$ is the estimated value, n is the number of samples.

**Fig. 2:**
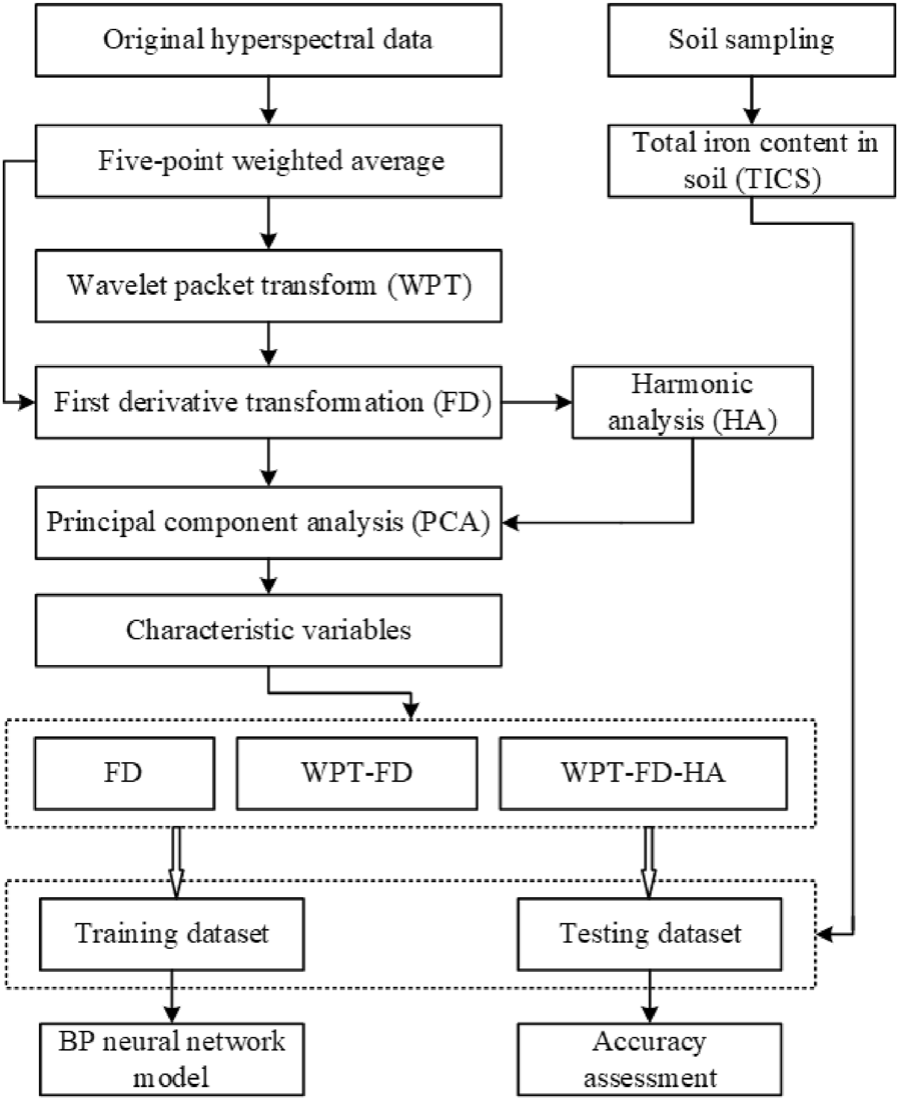
The flow chart of this study

To eliminate the spectral noise of the instrument, and process the HFI, the original spectral data were preprocessed by five-point weighted average, FD, WPT, and HA. The workflow is shown in Fig. 2.

## Results

### Spectral comparisons of different types of soil

**Fig. 3:**
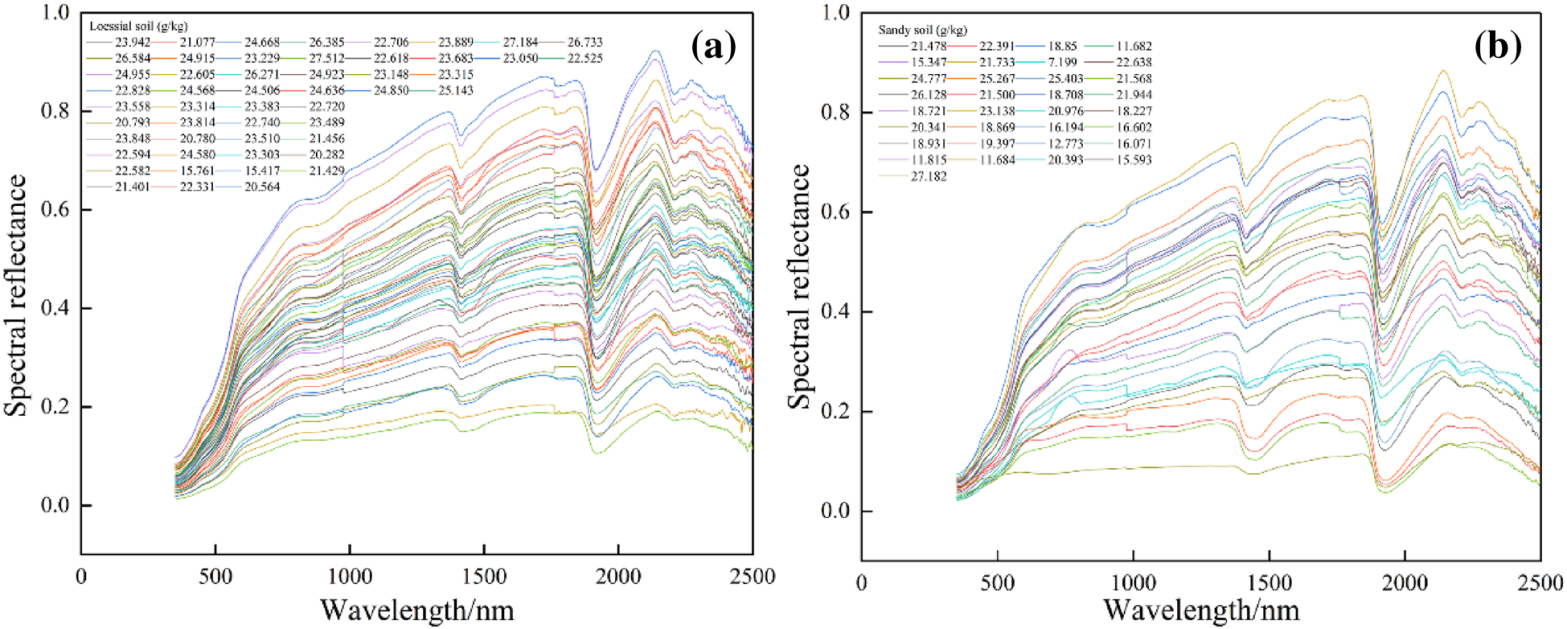
Primary spectral reflectance of different TICS: **a** loessial soil; **b** sandy soil

The hyperspectral curves of different types of soil were shown in Fig. 3. It can be seen that the waveform of different types of soil and soil with different iron content is generally similar. With the increase of TICS, reflectance did not show a significant increase or decrease trend, but the overall trend did not change (including the position of the absorption band). It indicates that the spectral reflectance cannot directly reflect the change of TICS and the new information needs to be mined.

### Characteristic bands selection

**Fig. 4:**
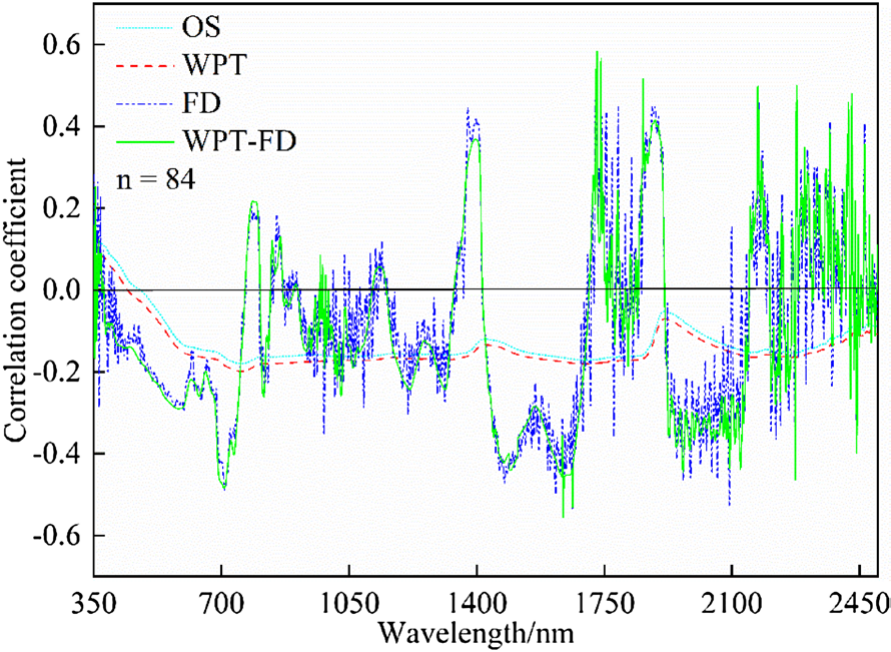
The correlation between original spectral data (OS), first derivative data (FD), wavelet packet transform data (WPT) and reconstructed first derivative data based WPT (WPT-FD) of two types of soils, and the TICS

Four kinds of data, including original spectral data (OS), first derivative data (FD), wavelet packet transform data (WPT), and reconstructed first derivative data based WPT (WPT-FD) were used to analyze the correlation with the TICS. The results are shown in Fig. 4.

The OS and WPT data were negatively correlated with TICS as a whole (except 350–500 nm) with the absolute value of the correlation coefficient (|r|) less than 0.2 (P  < 0.01). The correlation with FD, WPT-FD, and TICS was obviously increased, with the positive and negative values alternated. We calculated the correlation coefficients between FD, WPT-FD data, and their corresponding TICS at different bands to reduce the total number of bands for estimating TICS. The selection principles of characteristic bands were |r| > 0.55 and the number of selected bands kept moderate. Finally, 150 characteristic bands were selected from FD and WPT-FD data of all soil types. The number of bands remained the same as that of the single soil type to control the variables.

### Harmonic decomposition

**Fig. 5:**
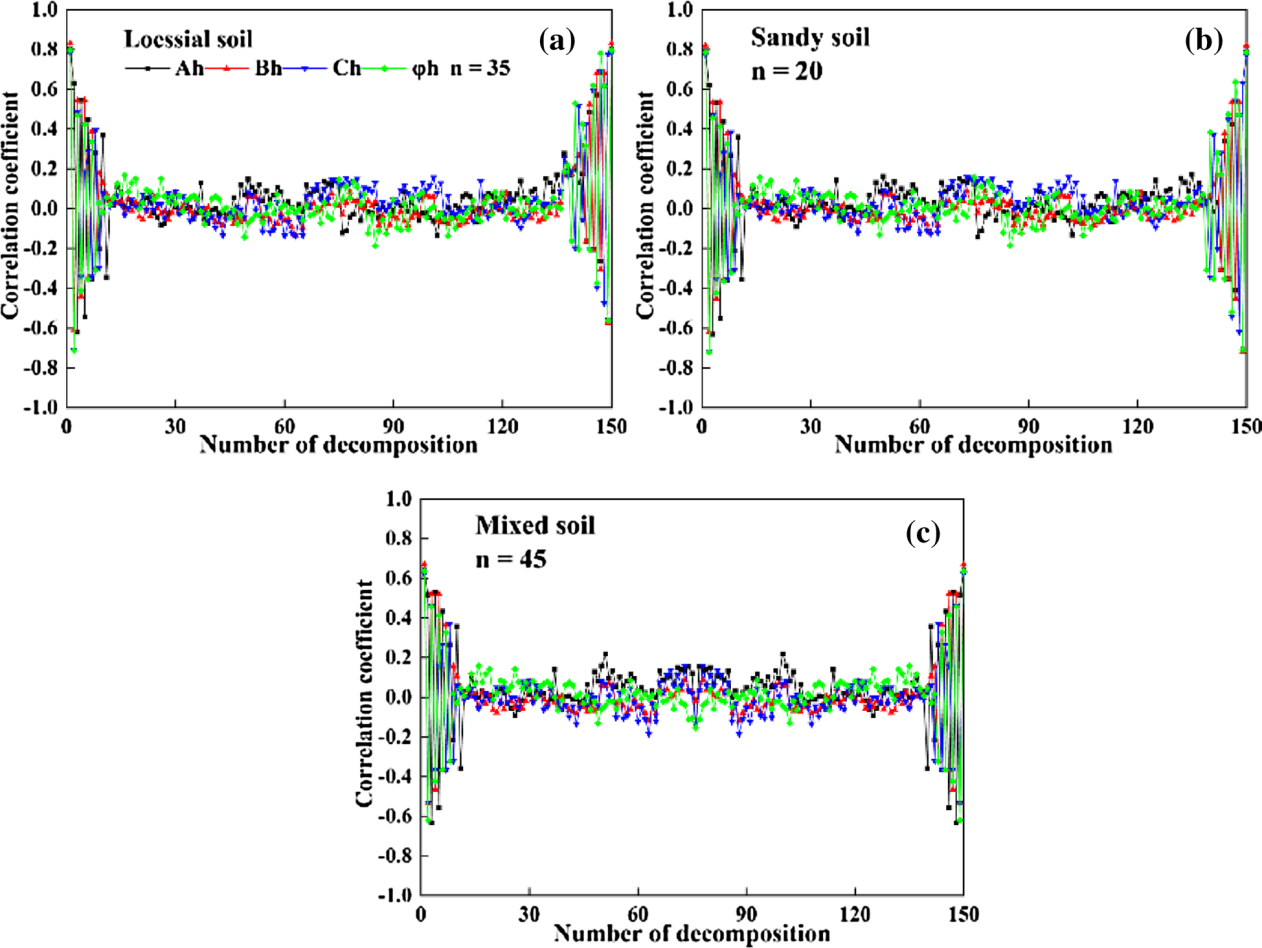
The correlation between the characteristic components of different harmonic energy spectra and TICS: **a** mixed soil; **b** loessial soil; **c** sandy soil

The WPT-FD data of loessial, sandy and mixed soils were decomposed by using eqs. (4)–(8) to obtain the characteristic components of harmonic energy spectra (A_0_/2, Ah, B_h_, C_h_, and φ_h_). The correlation coefficients between the harmonic components and TICS were calculated. The total number of bands of harmonic decomposition was 150. Considering the periodicity of sine and cosine functions, the times of harmonic decomposition were 150. Figure 5 shows the correlation coefficients between the characteristic components of different harmonic energy spectra and TICS of loessial soil, sandy soil, and mixed soil.

The results showed that there is a high correlation between harmonic components of first and latter several times and TICS, and the correlation shape of characteristic components (A_h_, B_h_, C_h_, and φ_h_ of harmonic energy spectra) were roughly axisymmetric (Fig. 5). At the beginning and end, the correlation between the HA components and TICS is more than 0.6 (P  < 0.01), and the other components show a weak correlation with TICS. Furthermore, there is the same performance in different types of soil, partly showing the robustness of HA parameters to TICS prediction. Therefore, the relationship between HA components and TICS can be well described by using half the parameters of an asymmetric graph. Another characteristic is periodicity: the correlation between HA components and TICS shows positive and negative alternation. Considering the periodicity and symmetry of the correlation between harmonic parameters and TICS, 12 harmonic characteristic parameters (A_0_/2, A_1_, A_2_, A_3_, B_1_, B_2_, B_3_, C_1_, C_2_, C_3_, φ_1_, and φ_2_) were selected combined with the correlation coefficients.

### Selection of characteristic variables for BP model

The spectral parameters and 12 harmonic characteristic parameters were analyzed by PCA to reduce the number of input layer neural networks and improve the network topology and estimation effect. For loessial soil, sandy soil, and mixed soil, the first five components were selected as input variables of BP models. The eigenvalue and variance contribution rate of PCA are shown in Table 2.

**Table 2:** Eigenvalue and variance contribution rate of different types of soil data

PCA	Soil types	Eigenvalue			Variance contribution (%)			Accumulative contribution (%)		
		FD	WPT-FD	WPT-FD-HA	FD	WPT-FD	WPT-FD-HA	FD	WPT-FD	WPT-FD-HA
PCA1	Mixed soil	6.91 × 10^–6^	3.62 × 10^–5^	1.46	74.82	84.01	90.00	74.82	84.01	90.00
PCA2		9.76 × 10^–7^	1.55 × 10^–6^	0.18	10.56	6.02	5.00	85.38	90.03	95.00
PCA3		4.36 × 10^–7^	4.26 × 10^–7^	1.64 × 10^–7^	4.72	4.11	2.00	90.10	94.14	97.00
PCA4		2.72 × 10^–7^	1.16 × 10^–7^	1.04 × 10^–7^	2.95	1.30	0.75	93.04	95.42	97.75
PCA5		1.26 × 10^–7^	4.55 × 10^–8^	6.93 × 10^–7^	1.36	0.12	0.23	94.41	95.56	98.99
PCA1	Loessial soil	3.39 × 10^–6^	1.56	2.82 × 10^–5^	77.00	88.81	95.88	77.00	88.71	95.88
PCA2		6.21 × 10^–7^	0.20	8.54 × 10^–7^	10.41	6.19	2.90	87.41	94.90	98.79
PCA3		4.85 × 10^–7^	0.07	1.28 × 10^–7^	6.15	2.09	0.43	93.56	96.99	99.22
PCA4		1.92 × 10^–7^	6.56 × 10^–4^	5.79 × 10^–8^	3.23	0.73	0.20	96.79	97.99	99.42
PCA5		1.70 × 10^–8^	3.64 × 10^–4^	4.56 × 10^–8^	0.86	0.51	0.16	97.65	98.50	99.57
PCA1	Sandy soil	9.11 × 10^–6^	4.71 × 10^–5^	1.34	86.94	89.27	90.75	86.94	89.27	90.75
PCA2		6.36 × 10^–7^	2.77 × 10^–6^	0.15	6.07	5.26	8.75	93.01	94.53	99.50
PCA3		2.12 × 10^–7^	1.86 × 10^–6^	6.65 × 10^–7^	2.02	3.51	0.26	95.03	98.04	99.76
PCA4		1.45 × 10^–7^	7.57 × 10^–7^	2.41 × 10^–7^	1.39	1.43	0.10	96.41	99.48	99.86
PCA5		1.01 × 10^–8^	8.32 × 10^–8^	1.61 × 10^–7^	1.05	0.16	0.05	97.46	99.64	99.90

For three types of soil (mixed soil, loessial soil, and sandy soil), the accumulative contribution rates of the first five principal components of WPT-FD data reached 95.56%, 98.50%, and 99.64%, respectively, which fully met the requirements of input variables of BP models (Table 2). The PCA results of WPT-FD data were better than those of FD data. The accumulative contribution rates of the first five principal components of WPT-FD-HA were 98.98%, 99.57%, and 99.90%, respectively, which basically contained the characteristic components of original harmonic energy spectra. Moreover, the results of WPT-FD-HA were better than those of WPT-FD, and the effects of data dimensionality reduction were also prior. Based on the above PCA results, three input variables of BP models (FD, WPT-FD, and WPT-FD-HA) were constructed. The PCA results of the WPT-FD-HA were the best, followed by WPT-FD, and the FD was the worst.

### Establishment of BP models and accuracy evaluation

**Fig. 6:**
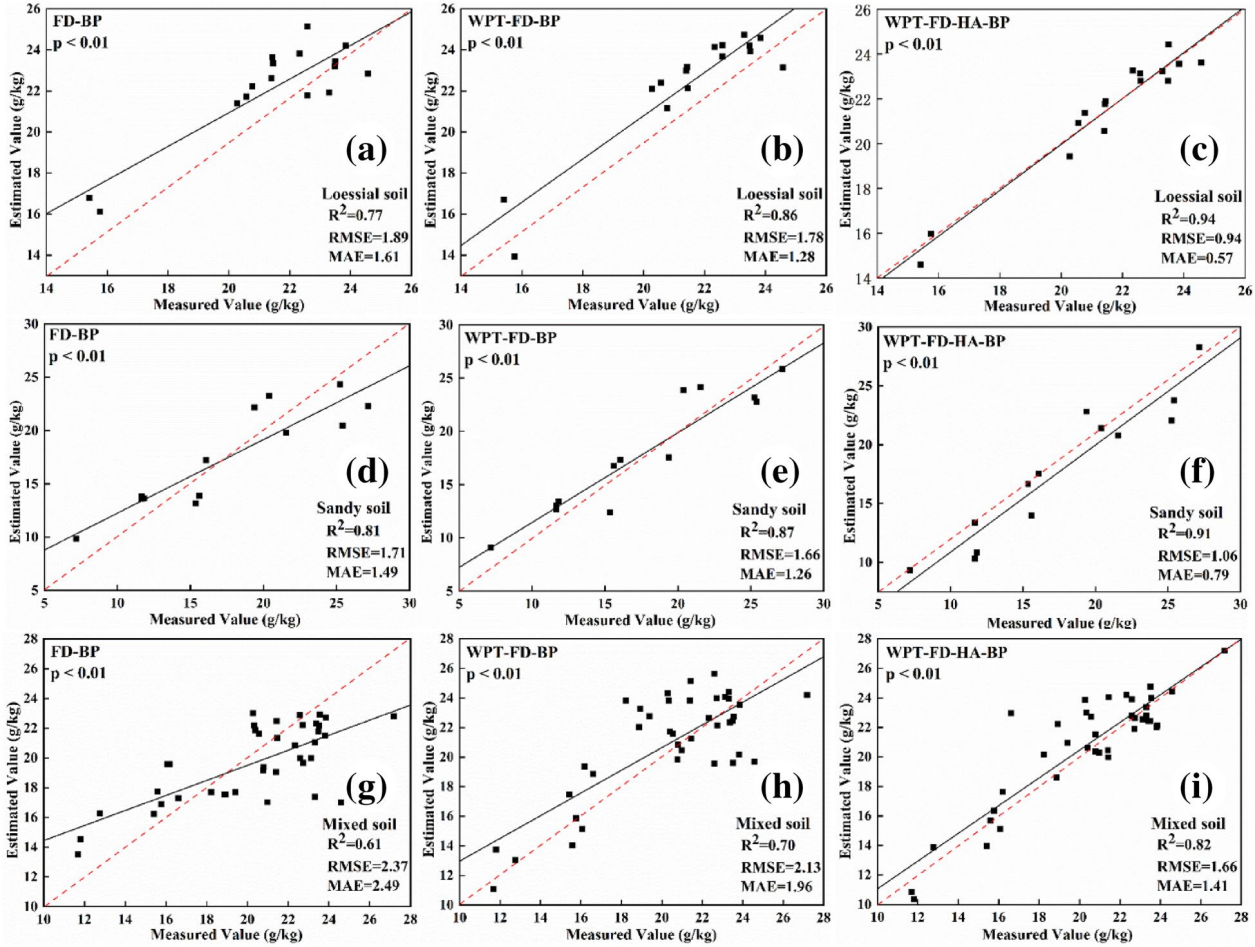
Validation models of TICS estimation of different types of soil: **a** FD-BP model of loessial soil; **b** WPT-FD-BP model of loessial soil; **c** WPT-FD-HA-BP model of loessial soil; **d** FD-BP model of sandy soil; **e** WPT-FD-BP model of sandy soil; **f** WPT-FD-HA-BP model of sandy soil; **g** FD-BP model of mixed soil; **h** WPT-FD-BP model of mixed soil; **i** WPT-FD-HA-BP model of mixed soil

BP estimation models of the TICS were constructed based on three types of variables (FD, WPT-FD, and WPT-FD-HA). Finally, three inversion models were established for three soil types: FD-BP, WPT-FD-BP, and WPT-FD-HA-BP. The inversion accuracy of different models for three soil types is shown in Table 3. Fitting results between estimated values and measured values of TICS of three types of soil are shown in Fig. 6.

**Table 3:** TICS estimation models of different types of soil

Soil types	BP models	R^2^	RMSE
Loessial soil	FD-BP	0.86	1.29
	WPT-FD-BP	0.92	1.16
	WPT-FD-HA-BP	0.95	0.68
Sandy soil	FD-BP	0.89	1.09
	WPT-FD-BP	0.93	0.88
	WPT-FD-HA-BP	0.94	0.71
Mixed soil	FD-BP	0.71	1.83
	WPT-FD-BP	0.79	1.57
	WPT-FD-HA-BP	0.87	1.11

For the three types of soil, the estimation result of the WPT-FD-BP model was evidently better than that of the FD-BP model (Table 3). The R^2^ of WPT-FD-BP models of different soils was 0.79 (mixed soil), 0.92 (loessial soil), and 0.93 (sandy soil), and RMSE was 1.57, 1.16, and 0.88, respectively. The R^2^ of the WPT-FD-HA-BP model was 0.87 (mixed soil), 0.95 (loessial soil), and 0.94 (sandy soil), which were higher than those of the WPT-FD-BP model. Moreover, the inversion accuracy of the WPT-FD-HA-BP model of a single soil type was higher than that of mixed soil.

Figure 6 showed the comparison between the retrieved and measured values of TICS of different types of soil. The performance of BP models under the conditions of single soil (loessial soil and sandy soil) type was better than that of the mixed soil, which indicated that there existed some differences in spectral characteristics and spectral characteristic bands of various soils. Therefore, the soil property information must be considered in the study of TICS estimation. In terms of the model structure, it can be seen that the best effects belong to the WPT-FD-HA-BP model, with the closest estimated values to the measured values. The artificial and parameter calculation errors in the process of selecting characteristic bands were reduced to some extent, making the newly constructed inversion factors more suitable for TICS estimation.

## Discussion

### Superiority analysis of harmonic analysis in parameters selection for TICS estimation

Soil spectrum is a comprehensive reflection of various soil properties, affecting by the factors of soil organic matter, soil color, soil texture, soil moisture, and mineral composition [28, 29]. For the complexity of the composition of soil spectrum, the performance of TICS estimation of characteristic variables obtained by traditional spectral transformation (first and second derivative, reciprocal, logarithm, and continuum removal methods) was often limited [30]. Moreover, compared with the organic matter, water content, and other constant elements, total iron content are obviously different. The main reason is that the low TICS results in a weak spectral signal, and the performance of the inversion models using variables obtained by conventional methods is poor [31].

Through the correlation analysis, it is found that the first derivative spectra tend to have a higher correlation with TICS than the original spectra (Fig. 4). The reason is that the spectral differentiation technology can partially eliminate the effects of atmospheric effects, environmental background, and shadows [32]. The bands with high correlation with TICS are mainly concentrated in the range of 1800–1900 nm (Fig. 4), consistent with the characteristic absorption bands of different types of soils (Fig. 3).

After WPT reconstruction, the WPT-FD data tend to perform better than the FD data on the correlation with TICS (Fig. 4). Many scholars also used the principle of WPT to denoise the original spectra in the process of spectral data processing for the estimation of soil water content and organic matter [33]. However, given the complex situation of TICS estimation, the correlation between the WPT-FD data with TICS needs to improve.

The HA method proposed in this paper can improve the correlation with TICS by using the harmonic components of harmonic decomposition (Fig. 5). Harmonic decomposition can transform spectral information into signal molecules, which is more stable than spectral parameters and can effectively improve the inversion accuracy of trace elements in the soil. Finally, 12 harmonic components whose |r| > 0.6 were determined. Based on the above spectral data preprocessing, we carried out the PCA method to eliminate the correlation between characteristic bands (characteristic parameters).

In general, the spectral preprocessing methods used in this paper may be suitable for other studies as well. The different types of these methods were combined and optimized to improve the feasibility of hyperspectral inversion. It provides technical support for the rapid estimation of TICS. According to the characteristics of different physical and chemical soil properties, selecting different methods to improve the accuracy of the model may be an important research direction for quantitative inversion of soil hyperspectral in the future.

### Improvement of model accuracy and universality by harmonic analysis

In similar studies, the estimation accuracy of the BP model in this paper (Table 3) is higher than the result of Xie et al. [34]. They used RBF neural network to estimate the TICS with the R^2^ of 0.70. It showed that the HA has a certain contribution to the optimization of BP input variables. For the three types of soils, the characteristic variables from WPT-FD-HA helped to significantly improve the estimation accuracy of TICS. It was more significant in the mixed soil, the TICS estimation accuracy of the WPT-FD-HA-BP model increased about 12%, but only 8% in loessial soil and 4% in sandy soil (compared with the WPT-FD-PCA-BP model), which indicated that the characteristic variables obtained by HA can effectively improve the inversion accuracy of total iron content in mixed soil. Furthermore, the BP model based on HA shows better performance than the model of conventional characteristic bands. Its advantage is that the harmonic parameters were obtained by the Fourier transform of the first derivative spectral data reconstructed by wavelet transform. The harmonic parameters replaced the conventional spectral characteristic parameters as the new model input variables to avoid the uncertainty of spectral parameters calculation. It concluded that the HA plays a decisive and stable role in the process of TICS estimation.

The BP estimation model based on HA can not only adapt to the inversion of total iron content in a single soil type but also has good performance for mixed soil (R^2^  = 0.87, RMSE  = 1.11), which reflects the effectiveness and feasibility of the TICS estimation using HA. For loessial soil and sandy soil, the WPT-FD-HA-BP model shows higher accuracy than the other two models (FD-BP and WPT-FD-BP), indicating that the WPT-FD-HA-BP model has better adaptability for single soil type of total iron content inversion (Table 3). For mixed soil, the inversion and validation accuracy of the WPT-FD-HA-BP model is much higher than that of the WPT-FD-BP model (Table 3; Fig. 6), which indicates that the harmonic parameters obtained by harmonic decomposition can effectively improve the TICS estimation, and the inversion variables can be well applied to the soil with rich types. The relationship between TICS and spectral reflectance is not a simple linear relationship [35], and a large number of studies have proved that the BP model is good in dealing with nonlinear problems [36]. Combined with the results of this experiment, the feasibility and superiority of the BP model in retrieving TICS were verified again.

As shown in Fig. 6, the WPT-FD-HA-BP validation models show the best performance (the highest R^2^, the lowest RMSE and MAE of different types of soil). Compared with the results of TICS estimation, the inversion accuracy in this paper is obviously improved. The results show that the new factors proposed in this present have some improvements compared with the traditional inversion parameters, which mainly act on the following aspects: (1) the spectral denoising effect of WPT is effective, which contributes to the optimization of BP inversion factors. (2) among the TICS estimation models of different types of soil, HA-based models were the best. This highlights the determinacy and stability of HA in the inversion process of total iron content. (3) the TICS estimation models based on HA can be well adapted to single soil and mixed soil types. (4) there is no single linear relationship between TICS and spectral reflectance, which verifies the feasibility and superiority of the BP model in the TICS estimation.

### Limitations and future work

Different types of soils were selected to monitor the complex soil environment. Although the preparation of artificial soil samples can help to obtain stable soil spectral data, it destroys the physical structure of soil and changes the optical characteristics of soil to a certain extent. Therefore, in the next study, we will focus on measuring more realistic soil spectral information to further verify the robustness and reliability of the method proposed in this paper. Remote sensing, as a non-destructive method, is promising in TICS estimation. A few studies show that hyperspectral data can accurately predict minerals. For example, multiple spectral bands were used to predict TICS by multiple linear regression, with R^2^ of 0.76 [11]. Combined with BP neural network, Shen et al. utilized some spectral transformation methods including FD, second-order differential, and continuum removal to retrieve the concentrations of iron and copper, with accuracy needing to be further improved [37]. With that, WPT and HA were employed to obtain the high precision inversion model of TICS. In the future, soil spectral data from more areas will be used to verify the usefulness of the proposed method.

## Conclusions

In this study, we proposed a kind of characteristic variable to estimate TICS using harmonic decomposition parameters. It is observed that the traditional spectral parameters are unstable and subject to noise in the TICS estimation. The HA transforms the spectra data from spectral-domain to frequency domain, which avoids the uncertainty and reduces the error of spectral parameter calculation. The HA-BP inversion models have a good performance in the application of estimating TICS for different types of soils. The WPT-FD-HA-BP model which is suitable for different soil types is a good inversion method of TICS and has certain universality. There is an important reference value for determining the best characteristic variable for the TICS estimation.

## Data Availability

The data presented in this study are available on request from the corresponding author.
